# Bacterial Alginate-Based Hydrogel Reduces Hydro-Mechanical Soil-Related Problems in Agriculture Facing Climate Change

**DOI:** 10.3390/polym14050922

**Published:** 2022-02-25

**Authors:** Cesar Barrientos-Sanhueza, Danny Cargnino-Cisternas, Alvaro Díaz-Barrera, Italo F. Cuneo

**Affiliations:** 1 Facultad de Ciencias Agronómicas y de los Alimentos, Pontificia Universidad Católica de Valparaíso, Valparaíso 2340025, Chile; c.barrientos.sanhueza@gmail.com (C.B.-S.); danycargnino29@gmail.com (D.C.-C.); 2 Escuela de Ingeniería Bioquímica, Pontificia Universidad Católica de Valparaíso, Valparaíso 2340025, Chile; alvaro.diaz@pucv.cl

**Keywords:** bacterial alginate, hydrogel, agriculture, soil hydro-mechanical behavior, climate change

## Abstract

Agricultural systems are facing the negative impacts of erosion and water scarcity, directly impacting the hydro-mechanical behavior of soil aggregation. Several technologies have been proposed to reduce hydro-mechanical soil-related problems in agriculture. Biopolymer-based hydrogels have been reported to be a great tool to tackle these problems in soils. In this study, we investigated the hydro-mechanical behavior of different soils media treated with Ca-bacterial alginate hydrogel. We used an unconfined uniaxial compression test, aggregate stability test and hydraulic conductivity measurements to investigate the mechanical and hydraulic behavior of treated soils media. Our results from unconfined uniaxial compression test showed that yield stress (i.e., strength) increased in treated soils with higher kaolinite and water content (i.e., HCM3), compared with untreated coarse quartz sand (i.e., CM1). Furthermore, we found that temperature is an important factor in the gelation capacity of our hydrogel. At room temperature, HCM3 displayed the higher aggregate stability, almost 5.5-fold compared with treated coarse quartz sand (HCM1), while this differential response was not sustained at warm temperature. In general, the addition of different quantities of kaolinite decreased the saturated hydraulic conductivity for all treatments. Finally, bright field microscopy imaging represents the soil media matrix between sand and clay particles with Ca-bacterial alginate hydrogel that modify the hydro-mechanical behavior of different soils media. The results of this study could be helpful for the soil-related problems in agriculture facing the negative effects of climate change.

## 1. Introduction

Poor aggregation of soil particles due to climate change (i.e., erosion and water scarcity) increase soil degradation, creating a negative feedback loop that threatens food production [1,2,3]. Soil structure governs soil physical behavior [1], which is key to support, sustain and improve plant production [4]. However, soil physical properties can be lost by (1) slaking, (2) dispersion, (3) mechanical disturbance and (4) compaction [4]. Slaking is the breakdown of aggregates at macroscopic level into small fragments [5], and soil aggregate resistance to slaking depends on the internal configuration of soil particles matrix, organic matter and cementation (e.g., bacterial EPS, hyphae, active humus and recently, biopolymers) [4,6,7]. While slaking is a macroscopic breakdown, dispersion is the total breakdown of aggregates into primary particles (i.e., clay, silt, sand and organic materials) [4,7]. Mechanical disturbance is the result of tillage and rainfall impact. Tillage is a common practice in crop production, which destroys soil structure and enhances wind and water erosion [8]. Finally, compaction is the result of applied external forces that can be natural (e.g., wetting-drying cycles) or anthropogenic (e.g., tillage and/or trafficking) [4,6]. Compaction prevents aeration and drainage, and promotes waterlogging, factors that strongly inhibit crop growth [6]. To prevent these negative impacts over agricultural soils subjected to the effects of climate change, several biopolymers’ materials (i.e., hydrogels) have been proposed in the past (e.g., gellan gum, chitosan, and alginate) [9,10,11,12].

Hydrogels in agriculture have been proved to be potential tools to modify the hydro-mechanical behavior of soils by (1) changing and improving the fluid mechanics of the fluid phase, (2) increasing the water-holding capacity and (3) improving the mechanical behavior of soils under different environmental stresses [12,13,14]. Hydrogels have been developed with the objective of generating the glue-like effects of natural biopolymers, such as root mucilage and bacterial EPS over agricultural soils [11,13,15]. In the process of gelation, temperature have been recognized as an important variable for the correct crosslinking of hydrogels due to its effect on the slow gelation that provides uniformity of the hydrogel matrix, and dramatically affects the mechanical properties of the resulting hydrogel [16,17]. Several types of biopolymers obtained from different sources (e.g., plants, microorganisms and even crustacean waste) have been implemented in the past [9,11,13,18,19]. Bacterial alginate is a particular biopolymer obtained through the cultivation and bioprocessing of the bacterium *Azotobacter vinelandii* [20]. It belongs to a wide range of linear chain polysaccharides with the capacity to create three-dimensional networks in the presence of several cations (e.g., Calcium, Potassium, Silica, and Aluminum) [11,12,21]. This linear chain polysaccharide is basically made of two monosaccharides: (1) 1→4 linked β-D- mannuronic acid; and (2) its C-5 epimer α-L-guluronic acid [21]. This type of material has been used in different human activities such as construction, biomedical engineering, the food industry and agriculture [10,11,19,20,22]. In our previous study, we used calcium as the covalent crosslinking agent to improve the ionic gel strength, and as a result the mechanical and hydraulic properties or coarse-quartz sand were greatly improved by adding the Ca-alginate hydrogel [7]. However, it is unclear whether these results could be expanded to different textured soils subjected to different conditions of water content and temperature.

Here, we aimed to study the effect of Ca-bacterial alginate hydrogel over the hydro-mechanical behavior of three types of soils composed by coarse quartz sand and kaolinite and subjected to different content of water and temperatures. We used unconfined uniaxial compression tests, falling head permeability tests, aggregate stability tests and bright field microscopy to analyze the mechanics and hydraulics of the different treatments. With this information, we try to provide information that could be used in agricultural systems that are facing the side effects of climate change (e.g., drought, wind erosion and heavy rainfall).

## 2. Materials and Methods

### 2.1. Soil Media

Three types of soil-porous materials with different particle size fractions were used in this study. The percent of fractions was estimated according to the dry weight of samples. Soils were composed by: (1) quartz sand (100% fraction); (2) quartz sand and kaolinite (95% and 5% fraction, respectively); and (3) quartz sand and kaolinite (90% and 10% fraction, respectively). To assemble the different soils, quartz sand (Migrin S.A., Algarrobo, Chile) was used, as it is highly susceptible to mechanical compressive stresses and highly permeable to water, mainly due to its lack of aggregate stability [6,23]. Secondly, kaolinite is a common clay present in many agricultural soils around the world, and is the most common mineral of the 1:1 type, with a planar diameter in the range of 0.1 to 2 µm, and a variable thickness of about 0.02–0.05 µm [6]. Besides, the unbalanced charges created by its structural network must be compensated by the adsorption of mainly cations from the surrounding solutions when it is wetted. Na^+^, K^+^, Mg^+2^, Ca^+2^ and Al^+3^ are the main cations adsorbed on the clay surfaces. These phenomena generate the cation exchange, a very important process in the soil solution that affects the nutrient balance and the flocculation-dispersion processes of soil colloids and soil aggregates [6]. A standard US sieve (C-tech instruments) was used to determine particle size distribution following the ASTM C136 protocol [24].

### 2.2. Cultivation and Bioprocessing of Bacterial Alginate from Azotobacter vinelandii

To recover bacterial alginate several stages were used. Bacterial strain of *Azotobacter vinelandii* ATCC 9046 (American Type Culture Collection, Manassas, VA, USA) was cultivated in a medium under atmospheric N fixation conditions, utilizing sucrose as carbon source. The fermentation stage was carried out in a 30 L bioreactor (Infors HT, Techfors, Rittergasse, Switzerland) at 300 rpm, 1 vvm, 30 °C and constant pH 7.0 controlled with 2 N NaOH [25]. 30 mL of culture broth were mixed with 3 mL of Na_4_EDTA at 0.1 M. and 3 mL of 1 M NaCl solutions. The final mix was centrifuged for 10 min at 7650 g. The alginate was recovered from the supernatant using cold propan-2-ol with a 1:3 volume, and then filtered with a 0.45 µm Millipore filter paper (MF-Millipore^®^) t. Later, the filtered precipitate was dried at 60 °C until reaching a constant weight. Then, the dried precipitate was ground and weighed again. The final solid product had a molecular weight (MW) of 453 ± 42 kDa [26]. Finally, to recover and store the final product, bacterial alginate was allowed to dry for 24 h at 60 °C in an oven (BOV-C30T, BioBase Industry, Shnagdong, China). The final dried bacterial alginate is ready for rehydration.

### 2.3. FTIR Spectroscopy Methodology to Quantify the Mannuronic and Guluronic Acid Contents in the Bacterial Alginate

A mix of techniques were implemented to quantify the mannuronic and guluronic acid contents of bacterial alginate obtained from cultivation and bioprocessing. In this case, we used Fourier transform infrared (FTIR) spectroscopy, accompanied with the total attenuated reflectance technique. To achieve the correct quantification of the mannuronic and guluronic acid, bacterial alginate samples were freeze-dried before FTIR measurements. Using a Jasco FT/IR-4600 spectrometer (Tokyo, Japan), several FTIR spectroscopy analyses were carried out from 500 to 4000 cm^−1^, until achieving sixty-four scans with a 4 cm^−1^ resolution [27]. Once the IR spectra were obtained, the stretching vibrations of COO– group, typical of bacterial alginate structures, were identified at 1612 and 1392 cm^−1^ [12]. Bonds of guluronic and mannuronic acid (i.e., stretching vibrations of CO–group) were recognized at 1314 and 1290 cm^−1^, respectively [12,28]. Ultimately, IR data was used to analyze and calculate the guluronic/mannuronic acid ratio using Bio-Rad software (version 18.3.1). Finally, the transmittance of the absorption band in 1320 and 1290 cm^−1^, guluronic/mannuronic ratio was calculated by division.

### 2.4. Sample Preparation

All the experiments were carried out in the Plant & Soil Biophysics and Biomechanics lab at the Pontificia Universidad Católica de Valparaíso. Six soil porous materials with triplicates were used in this study: CM1 = control consisting of quartz sand (100% fraction); CM2 = control consisting of quartz sand with kaolinite (95% and 5% fraction, respectively); CM3 = control consisting of quartz sand with kaolinite (90% and 10% fraction, respectively); HCM1 = consisting of quartz sand (100% fraction) mixed with Ca-bacterial alginate; HCM2 = consisting of quartz sand with kaolinite (95% and 5% fraction, respectively) mixed with Ca-bacterial alginate; and HCM3 = consisting of quartz sand with kaolinite (90% and 10% fraction, respectively) mixed with Ca-bacterial alginate.

### 2.5. Water-Soluble Solutions of Ca-Bacterial Alginate Hydrogel and Soil Mixture Conditioning

Dry bacterial alginate obtained from the cultivation and bioprocessing was hydrated to create a liquid solution. To promote the “eggbox” configuration of the bacterial alginate (i.e., the capacity to create three-dimensional networks in the presence of several cations), we used Ca(OH)_2_ as Ca^+2^ source. One of the best characteristics of Ca(OH)_2_ is its higher solubility in water compared with other Ca^+2^ sources (e.g., CaCO_3_, CaSO_4_, and CaO) [29]. Ca(OH)_2_ at 0.5 M, which is equivalent to 37.04 g L^−1^, was hydrated with filtered (FI) water using a magnetic stirrer (AccuPlate Hotplate Stirrer, Labnet, NY, USA), and the resultant solution with free Ca^+2^ molecules, released from the reaction, created the eggbox complex with the linear chain polysaccharide of the bacterial alginate (Supplementary Materials, Figure S1). Thus, Ca^+2^ was used as the covalent crosslinking agent to improve the ionic gel strength. Finally, to create a homogenized in-situ hydrogel, the liquid bacterial alginate was mixed with the Ca^+2^ solution and mixed with the different soils.

For the falling head permeability test and unconfined uniaxial compression test, the samples were prepared following the sample preparation methodology from [12]. First, for the falling head permeability test, 800 g of the different soils was weighed on plastic pots. The Ca-bacterial alginate hydrogel prepared previously was applied in liquid form (i.e., water-soluble solutions) over the different soils, and then mixed by hand with a spatula for 1 min to homogenize the samples. The bacterial alginate concentration was estimated according to the weight/weight (*w*/*w*) ratio of the soil. At 800 g of growing media, 0.8 g of bacterial alginate was applied in a water-soluble solution that is equal to 0.1% (*w*/*w*). For unconfined uniaxial compression test, samples of 90 g of the different soils were weighted in custom-made plastic devices that served as cylindrical molds (Supplementary Materials Figure S2). Second, the water volume of the tests applied was determined according to the saturation point of the different soils, which was 150 and 90 mL of water volume for the falling head permeability test and unconfined uniaxial compression test samples, respectively, according to previous tests in our lab. Finally, the curing time consisted of 3 days at two temperature conditions: (1) room temperature (20 °C); and (2) warm condition (30 °C) to evaluate the change of physical behaviors, and ensure that the treatments had the same reaction times with the different soil media [9,12,13].

### 2.6. Unconfined Uniaxial Compressive Testing

A texture analyzer (Model Ta.XT plusC, Stable Micro Systems Ltd., Surrey, UK) equipped with a compression plate of 100 mm of diameter (TMS 100 mm diameter, p/100) was used over cylindrical specimens of 2.8 cm in diameter × 5.5 cm height, with a cylindrical area of 6.1544 cm^2^ and a volume of 33.85 cm^3^. Before the compression test, the treatments were compacted in a custom-made mold attached to a lace curtain at the bottom to avoid soil loss by gravity (Supplementary Materials, Figure S3). Three levels of water content (WC) were used to test the mechanic behavior of the different soils, being: 100% (High); 75% (Medium); and 50% (Low). The water content was estimated gravimetrically. The texture analyzer software was set as follows: pre-test = 0.1 mm s^−1^, speed-test = 0.1 mm s^−1^, post-test = 0.01 mm s^−1^, force = 0.05 N. The trigger threshold was set in force, and the compression force was recorded in Newton (N) at 2 mm deformation to record with accuracy the elastic behavior of the treated soils. The data were then transformed into Mega Pascals (MPa) and axial strain.

**Figure 1:**
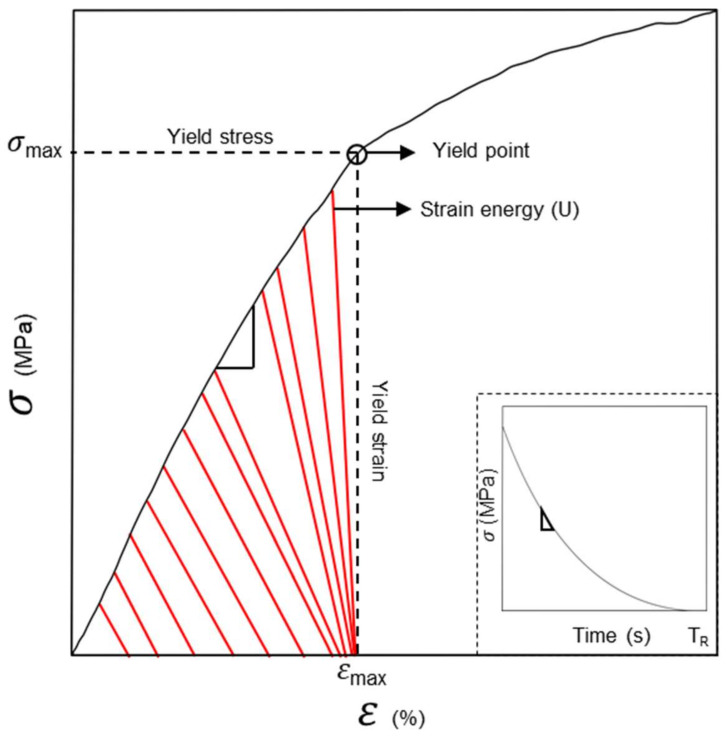
Representative figure of the curve elements used in the different calculus of soil media biomechanics. σ represents the stress (Force/Area) in MPa of the different treatments. ε represents the strain ($\frac{\Delta L}{L_{0}},\;\mathrm{where}\;L\;\mathrm{is}\;\mathrm{large}\;\mathrm{of}\;\mathrm{the}\;\mathrm{sample}$ ), which belongs to the sample deformation. E represents the Young´s modulus ($\frac{\sigma }{\varepsilon }$ ) in MPa, which represents the elastic zone of the different treated materials. Inlet, represent stress relaxation time (*T_r_*) for the materials once the mechanical disturbance starts to diminish.

The yield strength, yield strain, elastic modulus (Ε), rigidity modulus (G), strain energy (U) and relaxation time (*T_r_*) were obtained by the average of 3 repetitions per treatment (Figure 1). The Young’s modulus (Ε) was calculated using the slope elastic zone of the stress-strain curves under compression stress (*n* = 3) (Supplementary Materials, Figure S4). To calculate the viscous response component, the Niklas´s relaxation time equation (*T_r_*) [30] was used

(1) $$T_{r}=\frac{\sigma_{0}}{E\left(\frac{d\varepsilon }{\mathrm{dt}}\right)-\left(\frac{d\sigma }{\mathrm{dt}}\right)}$$

where $\sigma_{0}$ is the original stress (MPa), E is the Young´s modulus (MPa), $\frac{d\varepsilon }{\mathrm{dt}}$ is the strain at time t (s) and $\frac{d\sigma }{\mathrm{dt}}$ is stress (MPa) at time t (s).

To evaluate the distortion (i.e., rigidity) of the different elastic zones of soils, we can estimate the shear modulus (G) linking Ε and the Poisson´s coefficient (ν), as follows

(2) $$\;G=\frac{Ε}{2\left(1+\nu \right)}$$

where Ε is the Young´s modulus (MPa) and ν is the Poisson´s coefficient. In this case, due to the presence of hydrogel and different water content of soils, it is assumed that the different soil porous materials are incompressible, so that ν = 0.5 (ideal viscoelastic material) [6,31,32].

Finally, to estimate the amount of energy that must be introduced into the system (i.e., porous materials) to create deformation, the strain energy (U) was calculated [30], as follows

(3) $$U=\frac{\sigma \varepsilon }{2}V$$

where σ is stress (MPa), ε is strain and *V* is the volume of the material (m^3^). Besides, U can be estimated only for linear elastic materials. On the other hand, for non-elastic materials and plastic materials, U must be determined from the area under the stress-strain diagram (i.e., area under the curve).

### 2.7. Aggregate Stability Measurement

The pre-wetting method was used after the mechanical breakdown by shaking. Using the previous methodology, two experiments were carried out: the samples were subjected to (1) room temperature (i.e., 20 °C); and (2) a dry environment (i.e., 30 °C). This was done to evaluate the role of the temperature over the intra and inter-interactions in the different soil media. The experimental unit consisted of plastic pots of 250 g of growing media with four replications. In addition, the Ca-bacterial alginate hydrogel treatment was applied at a concentration of 0.1% (*w*/*w*) ratio of the soil porous material. For incubation, each experimental unit was exposed to 20 cycles (i.e., three weeks) of wetting and drying (dry wight). Wetting until saturation and drying parameters were defined gravimetrically. After incubation, mechanical breakdown by the hand shaking method were applied for each experimental unit. The samples were dried in the oven (BOV-C30T, BioBase Biodustry, Jinan, China) at 60 °C for 24 h, and then the soil media were screened by seven sieves (2000 µm, 1000 µm, 500 µm, 250 µm, 125 µm and 53 µm, respectively). After drying, the mass was recorded. Finally, the mean weight diameter (MWD) was calculated as an index of soil media aggregation using the following [5]

(4) $$\mathrm{MWD}=\overset{n}{\underset{i=1}{\sum }}X_{i}W_{i}$$

where MWD is (g/mm), X_i_ is the weight of dry growing media in the sieve i (g) and W_i_ is the average diameter of the pores of the adjacent sieve meshes (mm).

### 2.8. Hydraulic Conductivity Test

The falling head permeability method was implemented following the ASTM D5084-16a protocol [33]. After sample preparation, three consecutive falling head experiments (i.e., flushing events) were conducted on the different saturated soil media using the head permeability test set (HM-891, Gilson Company INC., Lewis Center, OH, USA). Four replications were used for this experiment, and the average of the three flushing events was used to compute saturated hydraulic conductivity (K) using an adaptation of Darcy´s law equation [34]

(5) $$K=\frac{\mathrm{aL}}{A\left(\Delta t\right)}\;*\;\mathrm{Ln}\left(\frac{h_{0}}{h_{1}}\right)$$

where a is the cross-sectional area of standpipe (m), L is the length of specimen (m), A is the cross-sectional area of specimen (m), ∆t is time elapsed (s) and h_0_ and h_1_ are the initial and final water meniscus heights of the water column (m). To verify the flow regime, we estimated the Reynolds number for each treatment as [12]

(6) Re=ρvDpμ

where ρ is the density of the fluid (kg m^−3^), v is the flow speed (m s^−1^), D_p_ is the diameter of particles of the growing media (mm) and μ is the dynamic viscosity of the fluid (Pa·s). Finally, to evaluate the relevance of the viscous forces versus surface tension of the Ca-bacterial alginate hydrogel filaments and its effects on the different composite materials, the Ohnesorge number was used [35,36]

(7) $$\mathrm{Oh}=\frac{\mu }{\sqrt{\rho \sigma L}}$$

where μ is the dynamic viscosity of the fluid (Pa·s), ρ is the density of the fluid (kg m^−3^), σ is the surface tension of water (N m^−1^) and L belongs to the length scale of filaments of Ca-bacterial alginate hydrogel between soil particle surfaces (m).

### 2.9. Bright Field Light Microscopy Imaging

To observe the internal matrix of the different soil-hydrogel composite materials, bright field light microscopy imaging was used. The eggbox effect of the Ca-bacterial alginate hydrogel matrix absorbs and retains the fluids that are in the solution. Thus, to identify the internal structures of the different composite materials, we used a blue ink-water solution to create contrast at the light source. Small samples of the different composite materials were fixed on glass slides and put under the microscope. Using a 200 µm micropipette, the blue ink-water solution with a gravimetric ratio of 1:2 was mixed with the soil samples [12,37]. Finally, to observe, identify and capture all the images, a Leica MC170HD digital camera attached to a Leica DMIL LED inverted microscope (Leica Microsystems, Wetzlar, Germany) was used.

### 2.10. Statistical Analysis

Analysis of variance (ANOVA) was performed using R version 4.0.0 statistical computing environment (R Core Team, 2021, R Foundation for Statistical Computing, Vienna, Austria) with the aid of the CAR software package [38]. The Shapiro–Wilk and Levene tests were used to check for the normality and homogeneity of variance, respectively. Tukey’s honest significant difference test was used to determine significant differences among treatments.

## 3. Results

### 3.1. Compression Test

**Figure 2:**
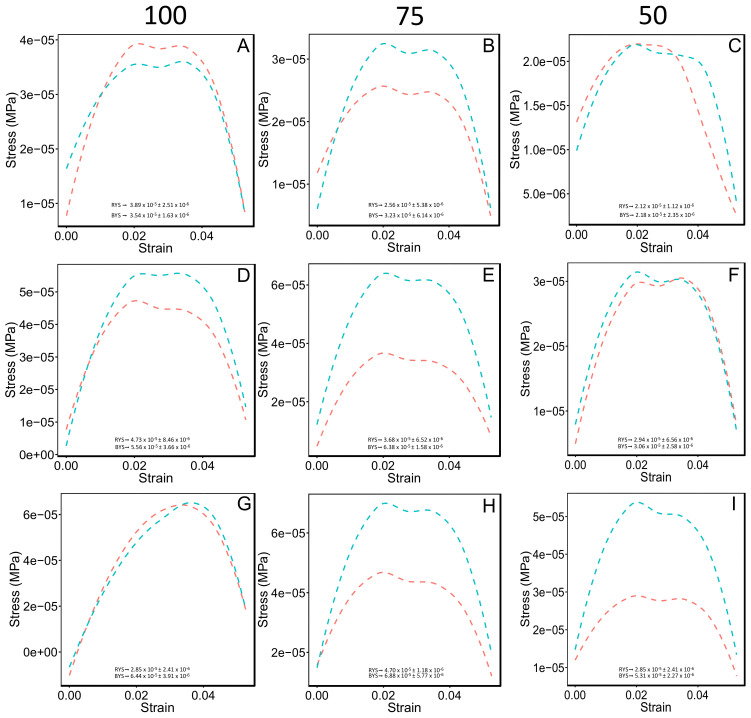
Unconfined uniaxial compression test (UCT) of the different treatments of the composite materials (i.e., CM1, CM2, CM3, HCM1, HCM2 and HCM3) showing the stress-strain (s-s) behavior. Figure shows three water content levels (i.e., 100, 75 and 50, respectively). Red segmented lines represent the soils media without the Ca-bacterial alginate hydrogel, and the blue segmented lines the soils media with Ca-bacterial alginate hydrogel. Respective red (RYS) and (BYS) yield stress curves are presented in (**A**–**I**) panels. Data are mean ± SE.

**Figure 3:**
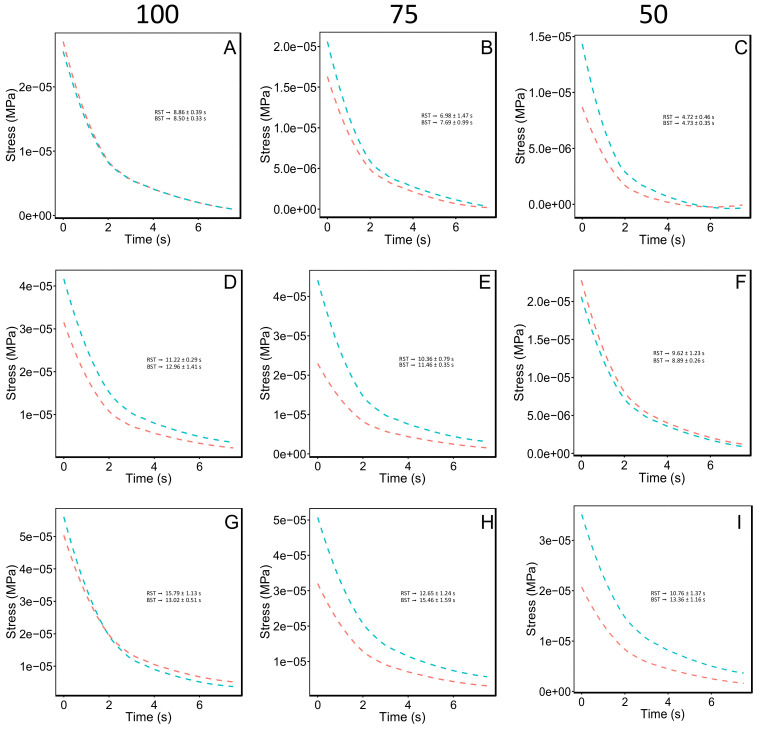
Stress relaxation curves (*T_rc_*) are presented for the different soil media treatments (i.e., CM1, CM2, CM3, HCM1, HCM2 and HCM3). Three levels of water content are shown (i.e., 100, 75, and 50, respectively). In Figure 2, red segmented lines represent the soils media without the Ca-bacterial alginate hydrogel, and the blue segmented lines the soils media with Ca-bacterial alginate hydrogel. Relaxation times (*T_r_*) are presented in (**A**–**I**) panels, being red (RST) and blue (BST) time relaxation. *T_r_* were calculated using the time relaxation equation [30] (See Equation (1)). Data are mean ± SE.

We used unconfined uniaxial compression test to quantify the mechanical behavior of the different soil porous materials on the stress-strain relationship, elastic properties, rigidity properties, strain energy and relaxation time. Significant differences were found in the stress and strain behavior among treatments (*p* = 6.98 × 10^−6^ and *p* = 2.20 × 10^−16^, respectively). HCM3 75 and 100 WC displayed a 3-fold higher yield stress compared with HCM1 50 WC and CM1 50 WC (Table S1, Figure 2). The rest of the treatments displayed similar values (Table S1, Figure 2). Strain values of the HCM3 100 WC treatment were 2.3-fold higher than that of CM3 75 WC, CM2 75 WC, CM1 75 WC, HCM1 50 WC and CM1 50 WC (Table S1, Figure 2). Significant differences were found in Young´s modulus (E) among treatments (*p* = 0.0002462). Young´s modulus (E) of HCM3 75 WC and HCM2 75 WC treatments showed higher values (3-fold higher) than the HCM1 100 WC and CM2 50 WC (i.e., lower values) treatments (Table S1). Regarding the shear modulus (G), significant differences were found among treatments (*p* = 0.0002462). HCM3 75 WC and HCM2 75 WC treatments displayed the higher Young´s modulus values compared to HCM1 100 WC and CM2 50 WC treatments (Table S1). Nevertheless, a big group of treatments had no statistical difference. The strain energy (U) allows us to classify and identify the elastic resilience (i.e., capacity of a body to absorb and subsequently release strain energy) [30] among treatments displaying statistical differences (*p* = 5.25 × 10^−13^). HCM3 100 WC showed the most elastic resilience, reaching 1.6-fold (4.76 × 10^−11^ ± 2.89 × 10^−12^ J SE) compared with CM3 100 WC (2.93 × 10^−11^ ± 4.49 × 10^−12^ J SE) (i.e., second treatment with most elastic resilience; Table S1). Finally, to estimate the viscous response component of different soil media, time relaxation (*T_r_*) was analyzed, and we found significant differences among treatments (*p* = 1.136 × 10^−6^; Figure 3). CM3 100 WC, HCM3 75 WC and HCM2 100 WC treatments showed the higher *T_r_* values (15.34 ± 0.88 s SE, 14.86 ± 1.60 s SE and 13.35 ± 1.26 s SE, respectively), compared with lower-value treatments CM2 50 WC, HCM2 50 WC, CM1 100 WC and HCM1 50 WC (9.69 ± 1.23 s SE, 9.00 ± 0.24 s SE, 8.88 ± 0.24 s SE, and 4.94 ± 0.42 s SE, respectively). These treatments consisted of soil media with 5% or without kaolinite, and low water content (i.e., 50%).

### 3.2. Aggregate Stability Test

**Figure 4:**
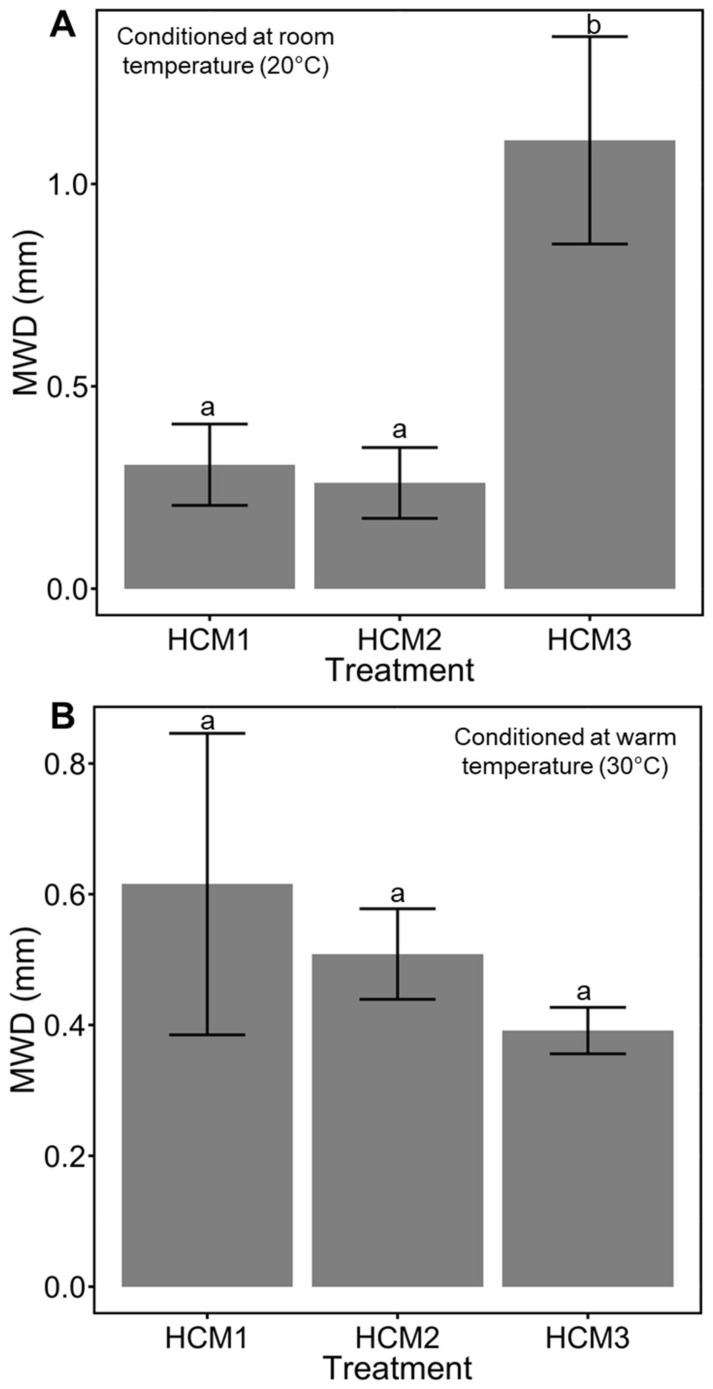
Stability of aggregates of the different treated soils media. Mean weight diameter (MWD) were obtained using Equation (3). (**A**), soil media treatments with Ca-bacterial alginate hydrogel conditioned at room temperature (20 °C). (**B**), soil media treatments with Ca-bacterial alginate hydrogel conditioned at warm temperature (30 °C). Data are means ± SE (*n* = 4). Mean followed by different letters are significantly different at *p* < 0.05 by Tukey´s test.

Mechanical breakdown/disruption of soil aggregates were used to test the aggregate stability of the different composite materials with Ca-bacterial alginate hydrogel. In previous studies, we evaluated the aggregate stability of coarse quartz sand with and without hydrogel [12]. In the present study, we measured the aggregate stability of different soil media with hydrogel under two environmental conditions: (1) room temperature (i.e., 20 °C); and (2) warm temperature (i.e., 30 °C). The environmental conditions were recreated in a growth chamber (Growth Chamber RGX-350, VMTECH, Huanghua, China). Low temperature had been recognized as an important variable for the correct crosslinking of hydrogels by the slow gelation that provides uniformity of the hydrogel matrix [16,17]. Under room temperature (i.e., 20 °C), HCM3 displayed the higher aggregate stability (1.11 ± 0.26 g mm^−1^ SE) almost 5.5-fold and 3.6-fold, compared with HCM2 (0.26 ± 0.08 g mm^−1^ SE) and HCM1 (0.31 ± 0.1 g mm^−1^ SE), respectively. Significant differences of aggregate stability were found among room temperature treatments (*p* = 0.009512) (Figure 4A). Nevertheless, the 30 °C condition produced a differential effect compared with the room temperature condition between the treatments (see Figure 4B). Higher temperatures could cause and increase Ca^+2^ reactivity [39]. Moreover, if the low critical solution temperature (LCST) is reached, a phase transition could occur [17]. HCM1, HCM2 and HCM3 showed similar aggregate stability behavior (0,61 ± 0.23 g mm^−1^ SE, 0.5 ± 0.06 g mm^−1^ SE and 0.39 ± 0.03 g mm^−1^ SE, respectively). No significant differences in aggregate stability were found among warm temperature treatments (*p* = 0.5012).

### 3.3. Hydraulic Conductivity Test

To quantify the effects of the Ca-alginate hydrogel over the different soil media, we used the falling head permeability test to measure saturated hydraulic conductivity (*K*). Soil composite materials with Ca-bacterial alginate hydrogel were subjected to the same environmental conditions of the aggregate stability test. As previously explained, the temperature plays a key role in the crosslinking of hydrogels. We measured how these two environmental conditions (i.e., room temperature and warm temperature) affected the saturated hydraulic conductivity of the different soils’ media. It is important to highlight that all treatments laid into Darcy´s flow regime (i.e., low Reynolds number Re ≈ 0.02). Furthermore, estimated Oh numbers of CM1, CM2, and CM3 were of ~0.16, representing the liquid bridges between the soil particles. HCM1, HCM2 and HCM3 displayed Oh numbers of ~83.4, caused by the incorporation of Ca-bacterial alginate hydrogel to the soil matrix.

**Figure 5:**
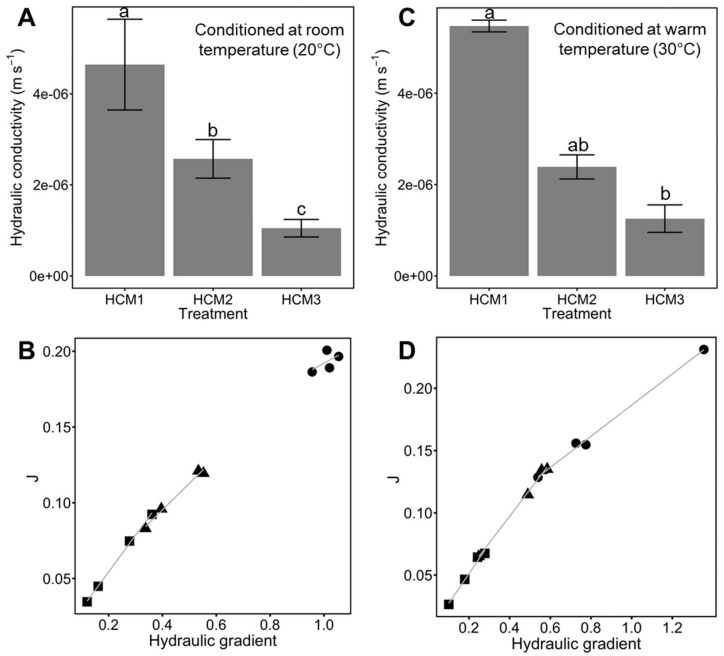
(**A**)Hydraulic conductivity (k) of the different composite materials with Ca-bacterial alginate hydrogel, at room temperature (i.e., 20 °C). (**B**), Shows the relationship of the flux density-hydraulic gradient of the different composite materials with Ca-bacterial alginate hydrogel, at room temperature. (**C**), Hydraulic conductivity (k) of the different composite materials with Ca-bacterial alginate hydrogel, at warm temperature (i.e., 30 °C). (**D**) Flux density-hydraulic gradient relationship of the different composite materials with Ca-bacterial alginate hydrogel, under warm temperatures. All treatments lay into Darcy´s model regime, which is explained by the low Reynolds number (Re ≈ 0.02). Circles, triangles and squares represent HCM1, HCM2 and HCM3, respectively. Data are means ± SE (*n* = 4). Mean followed by different letters are significantly different at *p* < 0.05 by Tukey´s test.

In general, the addition of different quantities of kaolinite decreased the saturated hydraulic conductivity (Figure 5). Under room temperature, HCM1 displayed the higher value (5.47 × 10^−6^ ± 1.28 × 10^−7^ SE m s^−1^), almost 2.3-fold and 4.4-fold compared with HCM2 (2.39 × 10^−6^ ± 2.64 × 10^−7^ SE m s^−1^) and HCM3 (1.25 × 10^−6^ ± 3.01 × 10^−7^ SE m s^−1^), respectively. HCM3 achieved to reduce *K* substantially, compared with HCM1 (77.1%) and HCM2 (56.3%). Significant differences of saturated hydraulic conductivity were found among room temperature treatments (*p* = 1.769 × 10^−6^) (Figure 5C,D). The 30 °C environment produced a similar hydraulic behavior as room temperature conditions. HCM1 had slight changes, with a reduction in *K* compared with HCM1 at room temperature (4.64 × 10^−6^ ± 9.97 × 10^−7^ SE m s^−1^ and 5.47 × 10^−6^ ± 1.28 × 10^−7^ SE m s^−1^). Additionally, HCM1 reached almost 1.8-fold and 4.5-fold compared with HCM2 (2.57 × 10^−6^ ± 4.24 × 10^−7^ SE m s^−1^) and HCM3 (1.05 × 10^−6^ ± 1.92 × 10^−7^ SE m s^−1^), respectively. However, HCM2 showed a reduction of *K* of 55.4% compared with HCM1. HCM3 showed the more prominent reduction of *K* (77.3%) compared with HCM1, and 40.5% compared with HCM2. Significant differences of saturated hydraulic conductivity were found among warm temperature treatments (*p* = 0.009856) (Figure 5A,B).

### 3.4. Bright Field Light Microscopy Imaging

**Figure 6:**
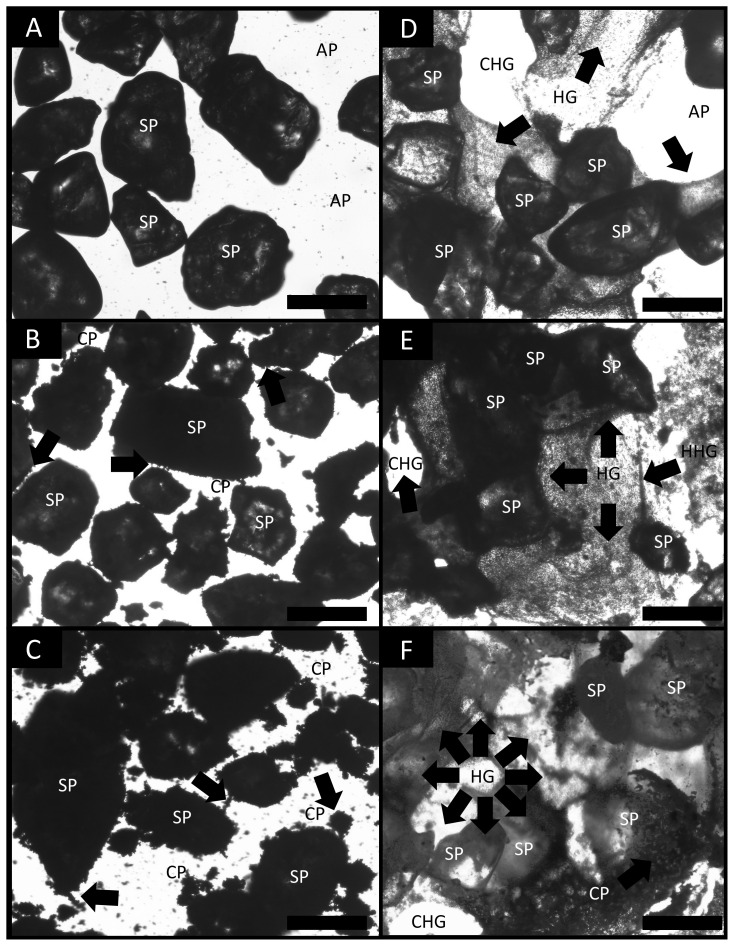
Bright field microscopy images of (**A**) CM1, (**B**) CM2, (**C**) CM3, (**D**) HCM1, (**E**) HCM2 and (**F)** HCM3. To visualize the different soil media with Ca-bacterial alginate hydrogel, a blue-in solution was used. Black and white color balance were used to enhance the contrast. All samples had a magnification rate of 5×. AP, air pocket; SP, sand particle; CP, clay particle; HG, Ca-bacterial alginate hydrogel; EHG, embolized Ca-bacterial alginate hydrogel; HHG, hollow cylinder of Ca-bacterial alginate hydrogel. Bars = 500 µm. (**A**–**C**) black arrows indicate clay particles over and around sand particles. (**D**–**F**) black arrows indicate the Ca-bacterial alginate hydrogel-sand particles interface, the formation of cavitated hydrogel (CHG) and the presence of a hollow cylinder hydrogel (HHG).

We used bright field light microscopy imaging to observe, identify and capture all the details of the different composite materials with Ca-bacterial alginate hydrogel (HCM1, HCM2 and HCM3). Composite materials without Ca-bacterial alginate hydrogel (i.e., CM1, CM2 and CM3) showed direct contact between the sand particles (Figure 6A), and the deposition of clay particles (CP) over the sand particles (SP) created several air pockets (AP) (Figure 6B,C).

Nevertheless, the addition of Ca-bacterial alginate hydrogel changes the structure of the composite materials at the particle level, showing a three-dimensional network indicated by black arrows (Figure 6D–F). Furthermore, cavitated hydrogel (CHG) structures were formed inside the Ca-bacterial alginate-composite material matrix by its high initial concentration. Finally, hollow cylinder hydrogel can be appreciated (Figure 6E).

## 4. Discussion

Availability of important belowground resources for plants such as water and nutrients deeply depend on soil physical properties that are currently threatened by the side effects of climate change phenomena (i.e., wind erosion, heavy rainfall and drought) [6]. In the present study, we provide a detailed depiction of the mechanics and hydraulics of different types of soils treated with Ca-bacterial alginate hydrogel, along with different content of water and temperatures. Our results highlight that Ca-bacterial alginate hydrogel can mechanically improve the yield stress of soils, and enhance the soil strength to mechanical disruption, in agreement with previous results [40]. Furthermore, the results presented here emphasized the importance of water content and temperature over the mechanics and hydraulics of the different treated soils.

To understand the mechanical behavior of the different soils treated with Ca-bacterial alginate hydrogel and different water contents, two types of mechanical measurements were carried out: (1) Unconfined uniaxial compression test (UCS); and (2) Aggregate stability test [5,12]. Between UCS test, several mechanical responses were measured and analyzed, such as: stress-strain relationship; elastic properties (E); rigidity properties (G); strain energy (U); and time relaxation (*T_r_*). The analysis of stress-strain relationship revealed that HCM3 75 and 100 WC had a strength value increased by 68%, compared with HCM1 50 WC and CM1 50 WC, the lower-yield stress treatments (Table S1). This data supports the idea that this hydrogel, based on bacterial alginate, can enhance the soil strength to mechanical disruption [40]. In this study, we also looked for the relaxation times (*T_r_*) measurement to have a better depiction of soil mechanics [30]. *T_r_* can exhibit different behavior; for example, if we are working with and ideal elastic material *T_r_* ≈∞, but if the material has a fluid like behavior *T_r_* ≈0 [30]. *T_r_* analysis is relevant to understand the shrinkage and swelling phenomena in soil media, since the change in volume of soil media is in function to its water content and the swelling properties of clay minerals [41,42]. Agricultural soils with ≤10% clay content are significantly affected by the shrink-swell phenomena [43]. The clay and water content define the hydromechanical behavior of *soil plasma* (i.e., clay matrix in the soil media), which alter the soil porosity and structural behavior under mechanical stress. Shrinkage and swelling phenomena cause crack forming in agricultural soils, generating problems with the soil structural resilience and the snapping of roots [42,44]. It is important to remark that the water content and physical stress behavior are highly non-linear processes, and differ between soil types [44]. In our study, we evaluated the effect of the different soil media with different water content and Ca-bacterial alginate hydrogel over the *T_r_* behavior curves. The results showed that CM3 100 WC displayed the higher value (15.34 ± 0.88 s SE), almost 4-fold compared with HCM1 50 WC (4.94 ± 0.42 s SE). For the same soil media but with Ca-bacterial alginate hydrogel (i.e., HCM3 100 WC), *T_r_* had a value of 12.59 ± 0.54 s SE, showing a reduction of 18% in *T_r_*. However, when the clay content in the soil media decreased from 10% to 5%, or the water content decreased from 100 to 75 or 50, the magnitude of *T_r_* decreases. *T_r_* behavior of different soil media with 10% clay content could be due to the small particle size (<2 µm), high surface area, which alters the internal structure of the different soil matrix, and greater water adsorption [45,46].

Temperature, as well as water content, changes the hydro-mechanical behavior of hydrogels within different porous media [10,16]. Higher temperatures could cause an increase in Ca^+2^ reactivity [39]. Moreover, if the low critical solution temperature (LCST) is reached, a Phase transition could occur [17]. In our study, two environmental conditions were used (i.e., room temperature and warm temperature) to test the hydraulic behavior of the different soil textures treated with Ca-bacterial alginate hydrogel. In our previous work, we measured the hydraulic conductivity of coarse quartz sand treated with bacterial alginate-based hydrogel, and found a decline of 33% in the hydraulic conductivity compared to non-treated sand [12]. Here, we observed that treatments HCM1, HCM2 and HCM3 had similar behavior at room and warm temperatures in terms of hydraulic conductivity. The high concentration of Ca-bacterial alginate hydrogel over the composite materials generates low Reynolds numbers (Re < 0.02; viscous forces domain over intertial forces), meaning that all treatments had a Darcy´s flow regime (i.e., laminar flow) [47,48]. Furthermore, for all treatments Oh >> 1, meaning that viscosity dominates over the inertial forces and fluid surface tension, leading to the formation of thicker filaments that hold the soil media matrix together (Figure 6D–F) [36]. The filaments act like the root mucilage ligands between soil particles, which could avoid the root-soil air gaps that are generated by drought. Moreover, the physical properties of bulk soil and rhizosphere, which involve soil structure and aggregation behavior, could be affected by using bacterial-alginate hydrogel [48]. The cohesion of soil particles, because of the filament bonding, could enhance the frictional bond and reduce the friction forces when a load is applied over the soil, improving the mechanical strength of the soil media [9,49]. However, the soil-hydrogel interaction depends on the water content of the specimen, and this may explain the differences observed among soil media treatments (Figure 2,Figure 3). Soil aggregation is another important property of soil physics that needs to be considered as part of the results when the bacterial-alginate hydrogel filaments bond soil particles. The rate of aggregation development and breakdown depends on the soil water content and soil temperature regime [50,51]. Climate change affects the soil water content and soil temperature regime by changing cycle patterns [52,53]. The stability of aggregates has a key role in the hydro-mechanical behavior of soils [54,55]. In this study, we evaluated the aggregate stability of different soil media using bacterial-alginate hydrogel under two environmental conditions (i.e., 20 °C and 30 °C). Our results highlight that environmental conditions of 20 °C can improve the aggregate stability of HCM3 by almost 5.5-fold and 3.6-fold compared with HCM2 and HCM1, respectively (Figure 4A). According to the aggregate stability classification system [5], HCM3 displayed very stable aggregates, compared with the very unstable aggregates of HCM1 and HCM2. Future studies should focus on trying to understand if these results persist under lower temperature treatments and under the influence of different pH.

Aggregate stability and hydraulic conductivity data shown here might be explained by the reinforcement mechanism of Ca-bacterial alginate hydrogel exerted among the soil particles. The mechanism is based on the strong adhesion of the biopolymer with the soil particles [9,56,57]. The physical structure of this reinforcement can be seen under bright field microscopy (Figure 6). In this study, we used coarse quartz sand and kaolinite to assemble the different soil media. The incorporation of Ca-bacterial alginate hydrogel in the different soil media changed the biophysical composition of soil particles at the microstructural level. Due to the high viscosity (µ) levels of Ca-bacterial alginate hydrogel (~0.501 Pa s), the viscous forces overpass inertial forces and fluid surface tension, which can alter the forces that holds water at macro and micropore levels in agricultural soils [36]. The effect of high µ creates embolized Ca-bacterial alginate hydrogel channels (CHG; Figure 6D). The appearance of multiple embolized Ca-bacterial alginate hydrogel channels could reorder the natural physical structures of the soil media. Finally, the use of different types of hydrogels based on the wide offer of polymers and biopolymers worldwide could be a useful tool to reduce the hydro-mechanical soil-related problems in agriculture facing climate change.

## 5. Conclusions

In this study, we investigated the hydro-mechanical behavior of different soils media treated with Ca-bacterial alginate hydrogel. Our results highlight that yield stress (i.e., strength) increases in treated soils with higher kaolinite and water content (i.e., HCM3), compared with untreated coarse quartz sand (i.e., CM1). We also found that temperature is an important factor in the gelation capacity of several hydrogels. At room temperature, HCM3 displayed the higher aggregate stability, at almost 5.5-fold compared with treated coarse quartz sand (HCM1). In general, the addition of different quantities of kaolinite, a typical clay particle found in different agricultural regions around the world, decreased the saturated hydraulic conductivity for all treatments. Further studies are still needed to come up with the best bacterial alginate-based hydrogel formulation, as well as experiments that test lower temperatures and pH during the gelation process. Furthermore, we think future studies should explore the interaction of these polymers with plants and microorganisms, and under stresses such as drought. Water scarcity and soil erosion are serious threats to humanity, and we believe the results from this study could be useful to growers and decision makers.

## Data Availability

The data presented in this study are available on request from the corresponding author.

## Supplementary Materials

The following supporting information can be downloaded at: https://www.mdpi.com/article/10.3390/polym14050922/s1: Result of the eggbox effect from the mix of the water-soluble solutions of rehydrated bacterial alginate and Ca(OH)*2*. Laminar flow cabinet were used to prepare the composite material samples without contamination of the solutions, Figure S1. Composite materials cylinders formed using cut-end falcon devices as molds. The dimensions of the cylinders were of D 2.8 cm × H 5.5 cm. (A), CM1, CM2 and CM3 cylindrical samples, respectively, and (B), HCM1, HCM2 and HCM3 cylindrical samples, respectively, Figure S2. Cut-end falcon device mold with sand in it. A fine porous mesh was glued at the end to avoid the particle loss due to gravity, Figure S3. Representative selection and estimation of the elastic zone of the different unconfined uniaxial compression test curves. Inlet, represent the statistical projection and selection of the slope in function of the yield stress point, Figure S4. Representative estimation of the strain energy of the different unconfined uniaxial compression test curves. The interior hatched represent the area under the curve, which is necessary for Equation (3), Figure S5. Falling head permeability test setup. (A) Conditioned composite material samples, ready to be used in the equipment. (B), Composite material sample inside in the container cylinder, ready to be connected to the water column and reservoir. (C), Container cylinder with the composite material inside connected to the water column and reservoir, ready to be flushed by DI water, Supporting information Figure S6.
