# Supplementary material for: Bacterial Alginate-Based Hydrogel Reduces Hydro-Mechanical Soil-Related Problems in Agriculture Facing Climate Change
Source: Polymers (Basel). 2022 Feb 25;14(5):922. doi: 10.3390/polym14050922 (PMC8912882; doi:10.3390/polym14050922)

Supplementary Materials

# Bacterial Alginate-Based Hydrogel Reduces Hydro-Mechanical Soil-Related Problems in Agriculture Facing Climate Change

Authors: Cesar Barrientos-Sanhueza, Danny Cargnino-Cisternas, Alvaro Díaz-Barrera, Italo F. Cuneo

The following Supporting Information is available for this article:

**Table S1.** Summarized statistical data of the mechanical properties of different treatments (n=3) after the unconfined uniaxial compressive testing. Data are means ± SE.

| Treatment | Yield stress (Mpa) | Yield strain | E (MPa) | G (MPa) | U (Joules) | Tr (s) |
|---|---|---|---|---|---|---|
| CM1 100WC | $3.89 \times 10^{-5} \pm 2.51 \times 10^{-6}$ | 0.019 | $2.02 \times 10^{-3} \pm 1.30 \times 10^{-4}$ | $1.51 \times 10^{-3} \pm 9.76 \times 10^{-5}$ | $1.27 \times 10^{-11} \pm 8.19 \times 10^{-13}$ | 8.88 ± 0.34 |
| CM1 75WC | $2.56 \times 10^{-5} \pm 5.38 \times 10^{-6}$ | 0.013 | $1.99 \times 10^{-3} \pm 4.19 \times 10^{-4}$ | $1.49 \times 10^{-3} \pm 3.14 \times 10^{-4}$ | $5.56 \times 10^{-12} \pm 1.17 \times 10^{-12}$ | 7.00 ± 1.49 |
| CM1 50WC | $2.12 \times 10^{-5} \pm 1.12 \times 10^{-6}$ | 0.008 | $2.77 \times 10^{-3} \pm 4.84 \times 10^{-4}$ | $2.08 \times 10^{-3} \pm 3.63 \times 10^{-4}$ | $6.37 \times 10^{-12} \pm 4.20 \times 10^{-12}$ | 6.69 ± 2.26 |
| CM2 100WC | $4.73 \times 10^{-5} \pm 8.46 \times 10^{-6}$ | 0.017 | $2.72 \times 10^{-3} \pm 4.86 \times 10^{-4}$ | $2.04 \times 10^{-3} \pm 3.64 \times 10^{-4}$ | $1.40 \times 10^{-11} \pm 2.50 \times 10^{-12}$ | 10.86 ± 0.34 |
| CM2 75WC | $3.68 \times 10^{-5} \pm 6.52 \times 10^{-6}$ | 0.014 | $2.59 \times 10^{-3} \pm 4.60 \times 10^{-4}$ | $1.95 \times 10^{-3} \pm 3.45 \times 10^{-4}$ | $8.83 \times 10^{-12} \pm 1.57 \times 10^{-12}$ | 10.31 ± 0.74 |
| CM2 50WC | $2.94 \times 10^{-5} \pm 6.56 \times 10^{-6}$ | 0.026 | $1.13 \times 10^{-3} \pm 2.53 \times 10^{-4}$ | $8.49 \times 10^{-4} \pm 1.90 \times 10^{-4}$ | $1.29 \times 10^{-11} \pm 2.89 \times 10^{-12}$ | 9.69 ± 1.23 |
| CM3 100WC | $6.31 \times 10^{-5} \pm 9.67 \times 10^{-6}$ | 0.027 | $2.30 \times 10^{-3} \pm 3.53 \times 10^{-4}$ | $1.73 \times 10^{-3} \pm 2.65 \times 10^{-4}$ | $2.93 \times 10^{-11} \pm 4.49 \times 10^{-12}$ | 15.34 ± 0.88 |
| CM3 75WC | $4.70 \times 10^{-5} \pm 1.18 \times 10^{-6}$ | 0.014 | $3.23 \times 10^{-3} \pm 8.08 \times 10^{-4}$ | $2.42 \times 10^{-3} \pm 6.06 \times 10^{-4}$ | $1.16 \times 10^{-11} \pm 2.91 \times 10^{-12}$ | 12.66 ± 1.26 |
| CM3 50WC | $2.85 \times 10^{-5} \pm 2.41 \times 10^{-6}$ | 0.016 | $1.81 \times 10^{-3} \pm 1.53 \times 10^{-4}$ | $1.36 \times 10^{-3} \pm 1.15 \times 10^{-4}$ | $7.59 \times 10^{-12} \pm 6.41 \times 10^{-13}$ | 10.83 ± 1.43 |
| HCM1 100WC | $3.54 \times 10^{-5} \pm 1.63 \times 10^{-6}$ | 0.026 | $1.37 \times 10^{-3} \pm 6.27 \times 10^{-4}$ | $1.02 \times 10^{-3} \pm 4.70 \times 10^{-5}$ | $1.56 \times 10^{-11} \pm 7.15 \times 10^{-13}$ | 8.50 ± 0.33 |
| HCM1 75WC | $3.23 \times 10^{-5} \pm 6.14 \times 10^{-6}$ | 0.016 | $2.00 \times 10^{-3} \pm 3.81 \times 10^{-4}$ | $1.50 \times 10^{-3} \pm 2.86 \times 10^{-4}$ | $8.80 \times 10^{-12} \pm 1.67 \times 10^{-12}$ | 8.15 ± 1.46 |
| HCM1 50WC | $2.18 \times 10^{-5} \pm 2.35 \times 10^{-6}$ | 0.010 | $2.08 \times 10^{-3} \pm 2.24 \times 10^{-4}$ | $1.56 \times 10^{-3} \pm 1.68 \times 10^{-4}$ | $3.87 \times 10^{-12} \pm 4.18 \times 10^{-13}$ | 4.94 ± 0.42 |
| HCM2 100WC | $5.56 \times 10^{-5} \pm 3.66 \times 10^{-6}$ | 0.023 | $2.46 \times 10^{-3} \pm 1.62 \times 10^{-4}$ | $1.84 \times 10^{-3} \pm 1.21 \times 10^{-4}$ | $2.13 \times 10^{-11} \pm 1.40 \times 10^{-12}$ | 13.35 ± 1.26 |
| HCM2 75WC | $6.38 \times 10^{-5} \pm 1.58 \times 10^{-6}$ | 0.017 | $3.75 \times 10^{-3} \pm 9.29 \times 10^{-4}$ | $2.81 \times 10^{-3} \pm 6.96 \times 10^{-4}$ | $1.84 \times 10^{-11} \pm 4.56 \times 10^{-12}$ | 11.74 ± 0.22 |
| HCM2 50WC | $3.06 \times 10^{-5} \pm 2.58 \times 10^{-6}$ | 0.015 | $2.03 \times 10^{-3} \pm 1.71 \times 10^{-4}$ | $1.52 \times 10^{-3} \pm 1.28 \times 10^{-4}$ | $7.82 \times 10^{-12} \pm 6.59 \times 10^{-13}$ | 9.00 ± 0.24 |
| HCM3 100WC | $6.44 \times 10^{-5} \pm 3.91 \times 10^{-6}$ | 0.044 | $1.48 \times 10^{-3} \pm 8.96 \times 10^{-5}$ | $1.11 \times 10^{-3} \pm 6.72 \times 10^{-5}$ | $4.76 \times 10^{-11} \pm 2.89 \times 10^{-12}$ | 12.59 ± 0.54 |
| HCM3 75WC | $6.88 \times 10^{-5} \pm 5.77 \times 10^{-6}$ | 0.017 | $3.95 \times 10^{-3} \pm 3.32 \times 10^{-4}$ | $2.97 \times 10^{-3} \pm 2.49 \times 10^{-4}$ | $2.02 \times 10^{-11} \pm 1.70 \times 10^{-12}$ | 14,86 ± 1.60 |
| HCM3 50WC | $5.31 \times 10^{-5} \pm 2.27 \times 10^{-6}$ | 0.015 | $3.60 \times 10^{-3} \pm 1.54 \times 10^{-4}$ | $2.70 \times 10^{-3} \pm 1.15 \times 10^{-4}$ | $1.33 \times 10^{-11} \pm 5.66 \times 10^{-13}$ | 12.81 ± 1.17 |

**Figure S1.** Result of the eggbox effect from the mix of the water-soluble solutions of rehydrated bacterial alginate and Ca(OH)2. Laminar flow cabinet were used to prepare the composite material samples without contamination.

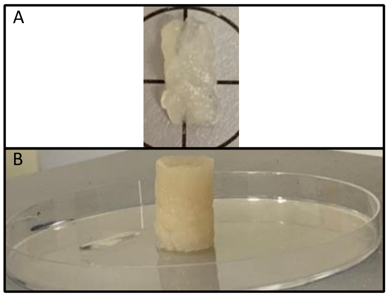


**Figure S2.** Composite materials cylinders formed using cut-end falcon devices as molds. The dimensions of the cylinders were of D 2.8 cm × H 5.5 cm. (A), CM1, CM2, and CM3 cylindrical samples, respectively. (B), HCM1, HCM2, and HCM3 cylindrical samples, respectively.

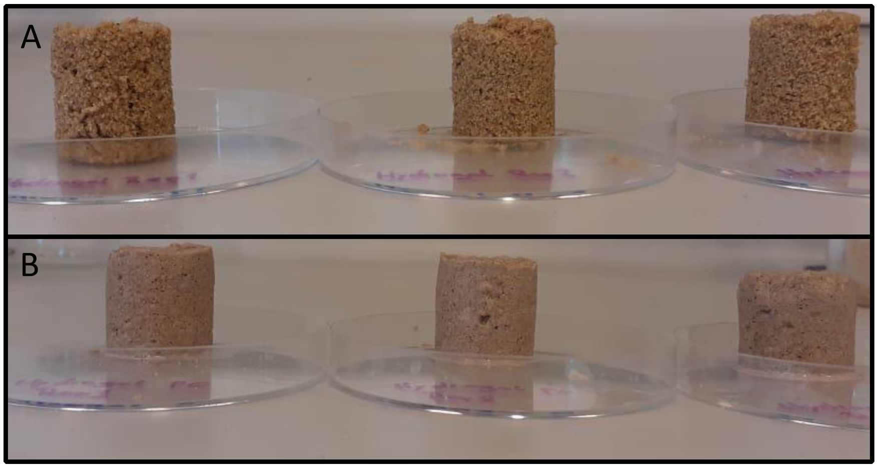

**Figure S3.** Cut-end falcon device mold with sand in it. A fine porous mesh was glued at the end to avoid the particle loss due to gravity.

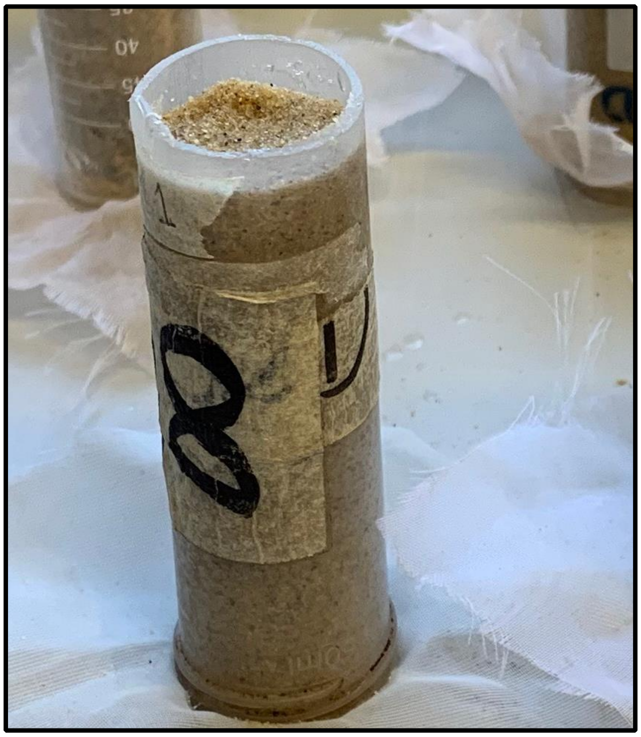

**Figure S4.** Representative selection and estimation of the elastic zone of the different unconfined uniaxial compression test curves. Inlet, represent the statistical projection and selection of the slope in function of the yield stress point.

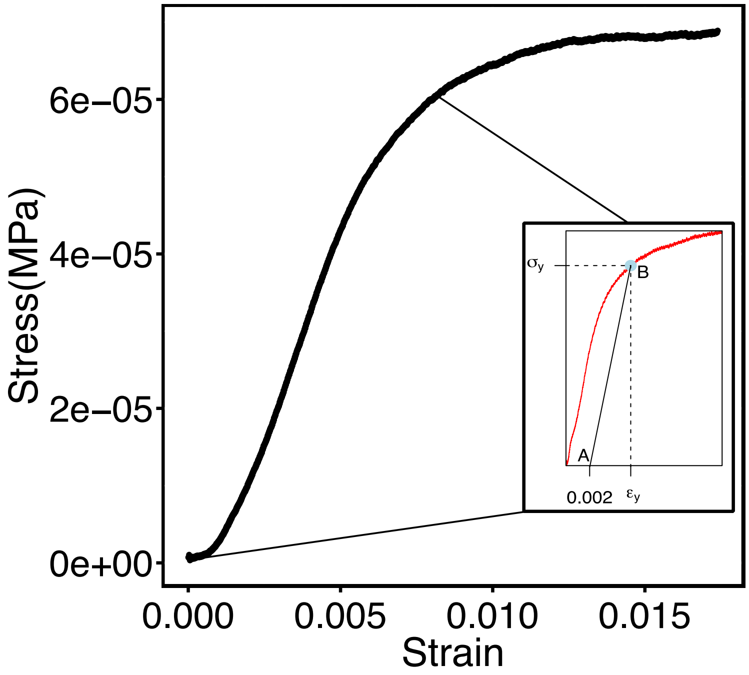

**Figure S5.** Representative estimation of the strain energy of the different unconfined uniaxial compression test curves. The interior hatched represent the area under the curve, which is necessary for the equation 3.

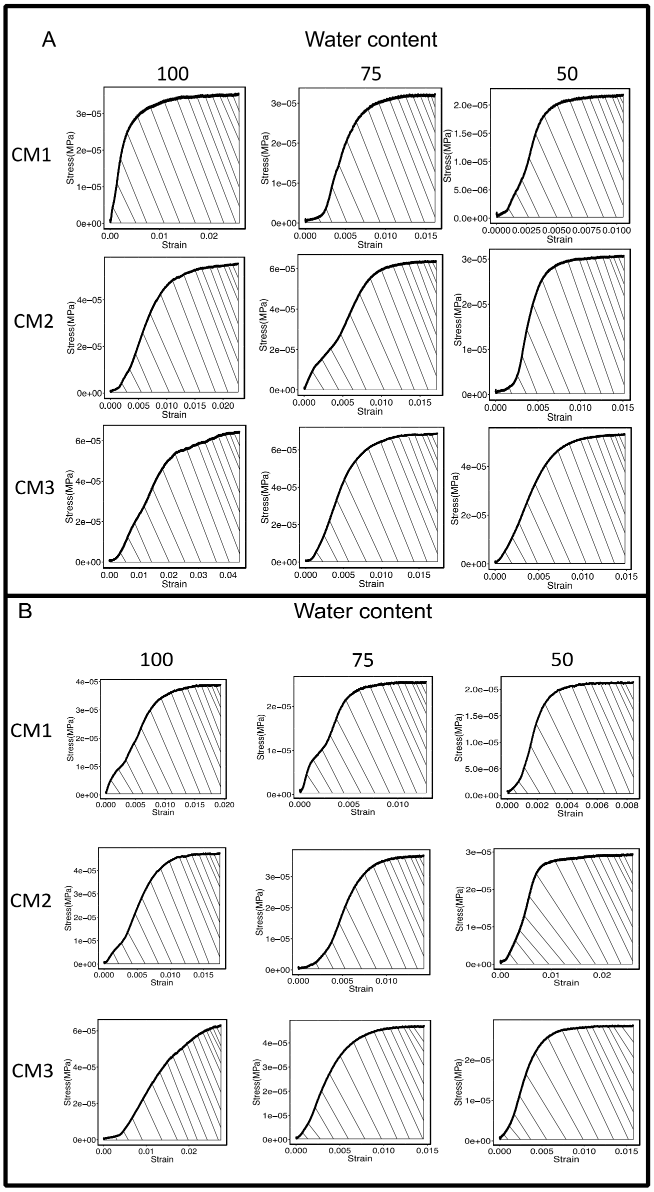

**Figure S6.** Falling head permeability test setup. (A) Conditioned composite material samples, ready to be used in the equipment. (B), Composite material sample inside in the container cylinder, ready to be connected to the water column and reservoir. (C), Container cylinder with the composite material inside connected to the water column and reservoir, ready to be flushed with FI (filtered) water.

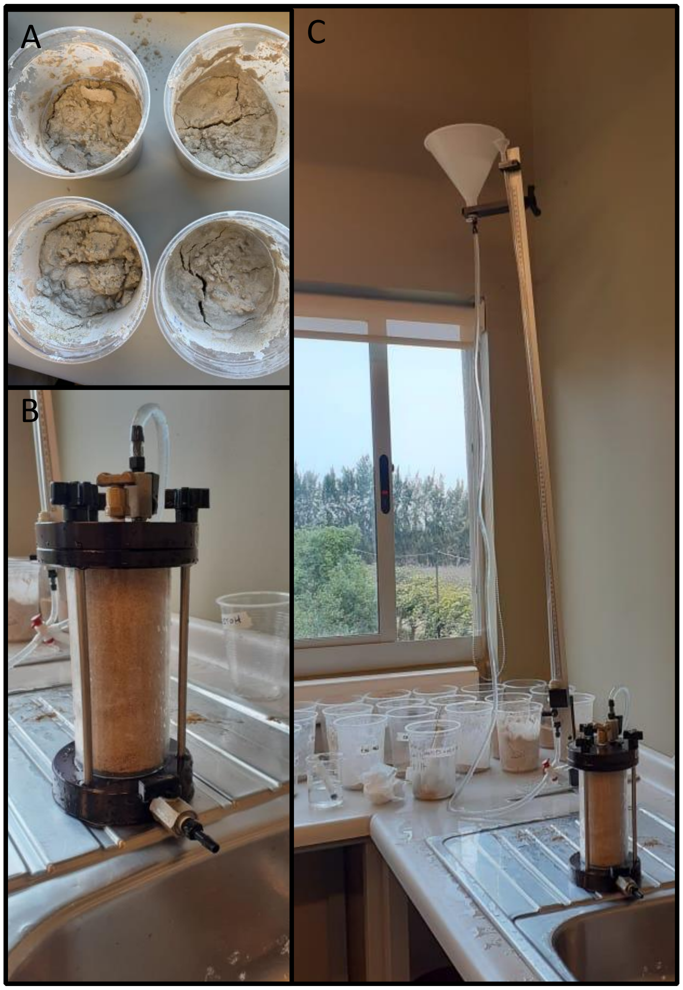

Supplement: Supplementary file 1 [file polymers-14-00922-s001.zip › polymers-1543599-SI.pdf]
